# Experimental Pharmacology of Glucosamine Sulfate

**DOI:** 10.1155/2011/939265

**Published:** 2011-10-09

**Authors:** Riccardo Chiusaroli, Tiziana Piepoli, Tiziano Zanelli, Paola Ballanti, Marco Lanza, Lucio C. Rovati, Gianfranco Caselli

**Affiliations:** ^1^R&D, Rottapharm SpA, 20900 Monza, Italy; ^2^Dipartimento di Scienze Radiologiche, Oncologiche e Anatomo-Patologiche, Policlinico Umberto I, Sapienza-Università di Roma, 00185 Roma, Italy

## Abstract

Several clinical studies demonstrated that glucosamine sulfate (GS) is effective in controlling osteoarthritis (OA), showing a structure-modifying action. However, little is known about the molecular mechanism(s) by which GS exerts such action and about the effects of GS at a tissue level on osteoarthritic cartilage and other joint structures. Here we provide mechanistic evidence suggesting that in vitro GS attenuates NF-**κ**B activation at concentrations in the range of those observed after GS administration to volunteers and patients, thus strengthening previous findings. Furthermore, we describe the effects of GS at a tissue level on the progression of the disease in a relevant model of spontaneous OA, the STR/ort mouse. In this model, the administration of GS at human corresponding doses was associated with a significant decrease of OA scores. Histomorphometry showed that the lesion surface was also significantly decreased, while the number of viable chondrocytes within the matrix was significantly increased. GS improved the course of OA in the STR/Ort mouse, by delaying cartilage breakdown as assessed histologically and histomorphometrically.

## 1. Introduction

Glucosamine sulfate (GS) is used all over the world for the therapy of osteoarthritis. Several clinical studies have shown that it is effective in controlling osteoarthritis (OA) not only in terms of symptoms, but also in reason of its ability to delay the progression of the disease, at least as far as can be assessed with the currently available clinical readouts [1–8]. However, there is still considerable confusion and conflict as to the real effects of glucosamine as an osteoarthritis disease modifying drug—in its heterogeneous preparations and different salts. Outcomes of the clinical trials have not been unanimous in assigning efficacy to glucosamine, due to a number of issues [9, 10]. Nevertheless, GS is recommended in 6/10 existing guidelines for the management of hip or knee OA [10].

Parallel to the clinical, a considerable amount of preclinical, experimental pharmacology studies have been accruing through the years, both by other groups and by ours, that contribute to the understanding of the modes of action of GS, although much certainly is yet to be done. Most of the efforts in this field were accurately reviewed by Block et al. [11]. It appears that the studies on glucosamine were mainly performed along two lines of research, one that employed mostly *in vitro* and cell biology methods, aimed at shedding light on the molecular mechanism, or mechanisms, through which glucosamine exerts its actions and the other, aimed at a thorough elucidation of the effects of GS, or other salt preparations, at a tissue level using *in vivo *approaches. Again, the differences in glucosamine salts used, in *in vitro* concentrations as well as in animal protocols, have sometimes led to confusing results. In the present study we have tried to contribute to clarification of such issues by applying what we believe are the most appropriate *in vitro* and *in vivo* protocols using standardized in vitro culture systems, a well-characterized animal model of OA pathology, a therapeutically relevant preparation and concentrations of GlcN salts, and standardized outcome measures, as pointed out by Block and colleagues [11].

Among the major factors hindering the interpretation of the results obtained with glucosamine across cell culture studies are the different concentrations used and the amount of glucose contained in the culture medium that could compete with glucosamine uptake. Here, we studied the effects of GS on the expression of several inflammation and matrix degradation factors, as assessed by quantitative real-time PCR on a relevant chondrocyte model, at concentrations found in plasma of human subjects after oral administration [12, 13] and in a medium containing physiological monosaccharide concentrations (5 mM) and in which galactose substitutes for glucose, thus facilitating glucosamine uptake in chondrocytes.

On the other hand, animal studies with glucosamine are also performed using different salts, doses, and animal species, and employing surgical induction of OA, while human OA is mostly spontaneous, idiopathic, and age-related. STR/ort mice spontaneously develop genuine OA with age; the whole joint undergoes degenerative changes very much like human OA, that consist in articular cartilage peeling, clefting, fibrillation, erosion; subchondral bone sclerosis with osteocyte necrosis; focally, fatty involution of the epiphyseal bone marrow; synovial hyperplasia; chondro-osseous metaplasias in capsula, ligaments, periosteum, leading to chondrophyte and osteophyte formation with ectopic bone development [14] and own data. Besides, STR/ort mice display obesity, hyperlipidemia, and hyperinsulinemia [15–17], all features that constitute risk factors for OA for humans. For all these observations, the clinical picture of the STR/ort mice closely resembles that of a typical human OA patient, so far as is known about either. Here we assessed the effects of GS on the course of OA in this mouse strain at a tissue level.

## 2. Materials and Methods

### 2.1. Materials

Crystalline glucosamine sulfate (GS) used is from Rottapharm (Monza, Italy). Leibovitz's Medium (containing 5 mM galactose), Dulbecco's phosphate-buffered saline (PBS), and Trypsin/EDTA were purchased from Invitrogen (CA, USA). Recombinant human Interleukin-1*β* (IL-1*β*, 100.000 U/mL, 2 *μ*g/mL) was purchased from Roche (IN, USA). U0126 was purchased from Cell Signaling (MA, USA) and SB242235 from Sigma (MI, USA).

### 2.2. Cell Culture

SW1353 cells (human chondrosarcoma cell line from ATCC, Promochem, UK) were grown adherent in Leibovitz's medium supplemented with 10% FBS and gentamicin (50 *μ*g/mL), at 37°C without Co_2_. Confluent cells were synchronized by incubating with Leibovitz's 0,4% FBS for 16 h. Cells were then pretreated for 1 h with GS 0,1–100 *μ*M for expression analysis of inflammatory and matrix degradative markers, and with GS 0,001–100 *μ*M for expression analysis of transcription factors subunits. The pre-treatment was followed by stimulation with IL-1*β* 2 ng/mL or IL-1*β* 10 ng/mL for expression analysis of inflammatory and matrix degradative markers or transcription factors subunits, respectively. Stimulation treatment with IL-1*β* lasted for 1, 2, 6, or 24 h depending from each gene kinetics of induction. Pretreatment and stimulation were performed in Leibovitz's 0,4% FBS.

### 2.3. Total RNA Purification

Total RNA was purified using ABI Prism^TM^ 6100 Nucleic Acid PrepStation, an RNA isolation platform from Applied Biosystems (Foster City, CA). This method uses a vacuum system that allows a reproducible purification without contaminations. Purified RNA was stored at −80°C.

### 2.4. Reverse Transcription (RT)

Total RNA was retro-transcribed with the High-Capacity cDNA Archive Kit (Applied Biosystems), adding 50 *μ*L of total RNA to 50 *μ*L of reaction mix. The reaction (final volume: 100 *μ*L) was carried out in a BioRad (Hercules, CA) “iCycler.”

Quantitative PCR (Real-Time PCR): Real-Time PCR is based on the quantitative relationship between the starting amount of the target sequence and the quantity of the PCR product obtained at each reaction cycle. Specific human and rat probes and primers used were purchased from Applied Biosystems as TaqMan Gene Expression Assays, while the human probe and primers of the endogenous control GAPDH (Glyceraldehyde-3-phosphate dehydrogenase) were PDARs (Pre-Developed TaqMan Assay Reagents). The reaction was performed using the ABI PRISM^®^ 7000 Sequence Detection System. Data analysis, normalized according to the amplified values of GAPDH, was done with the aid of the Relative Quantification/RQ Software (Applied Biosystems). Each sample was analyzed in triplicate.

### 2.5. Statistical Analysis

For each experiment, percentage effect of GS on gene expression induced by IL-1*β* was calculated starting from the relative quantity data obtained with the Relative Quantification/RQ Software (Applied Biosystems). 

### 2.6. Animal Studies

For our purposes, Harlan Italy developed a breeding colony of STR/ort mice. In order to double-check that the phenotype was still consistent with what described [14], we analyzed histologically mice from different groups of age: 4, 5, 6, 7, 8, and 9 months old, *n* = 20–24 (data not shown). In our hands, 100% of 5-month-old male STR/ort mice presented some degree of OA. Therefore, as we aimed for a curative protocol, and not a preventive one, we decided to enroll mice in each study as they reached 5 months of age (*n* = 20–22). One group of 5-month old mice was used as a “baseline” control. Glucosamine sulfate (Rottapharm) was administered subcutaneously dissolved in saline at the doses of 200 and 400 mg/kg. Mice were randomized for treatment, treated once daily for 3 months, and euthanized at the end of the treatment with collection of tissue (both hind limbs) for histology. Several nonconsecutive sections from each knee were stained with toluidine blue and blind scored according to both Mankin's and the OARSI method. Histomorphometry was also performed using the Osteomeasure image analysis system (Osteometrics, Atlanta, GA), with the operator still blinded to the experimental groups. Histomorphometric parameters analyzed were (i) lesion surface and (ii) number of viable chondrocytes within the articular cartilage. All scoring and measurements were performed on the medial tibial compartment of the knees. Statistical analysis was ANOVA followed by Dunn's or Dunnett's tests comparing all treatment groups versus vehicle.

## 3. Results

### 3.1. Cell Studies

As isolated primary chondrocytes have little proliferation capacity and tend to dedifferentiate to fibroblast-like cells in culture, we used a human chondrosarcoma cell line (SW1353) as a well-established chondrocyte model. We started performing time course experiments to determine the optimal IL-1*β* stimulation time for each parameter analyzed (data not shown). The experiments have been performed three times with comparable results. In this paper, we show representative experiments done at the optimal stimulation time for each transcript.

#### 3.1.1. GS Effect on Inflammatory Markers

**Figure 1:**
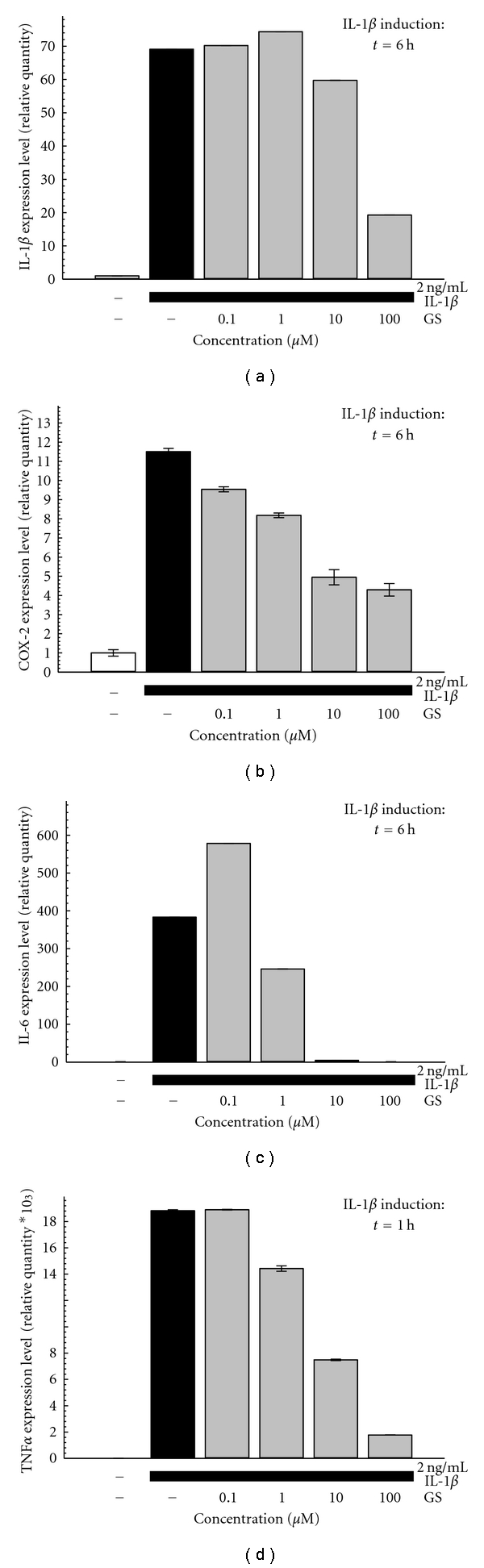
Effect of GS on IL-1*β*-induced gene expression of IL-1*β* (a), COX-2 (b), IL-6 (c), and TNF*α* (d). One hour after the pretreatment with GS (0,1–100 *μ*M), SW1353 are stimulated with IL-1*β* (2 ng/mL) as indicated. Representative pictures of 3 to 5 independent experiments are shown. Data are mean ± SD of three replicates.

We analyzed some inflammatory markers commonly involved in the OA disease. IL-1*β* induced the expression of all the inflammatory marker analyzed. The study of COX-2 was done after 6 h of stimulation, and in these conditions GS was effective in reducing its expression with a minimal effective concentration (MEC) of 1 *μ*M. The other inflammatory markers considered in our study are cytokines. The local production of cytokines is strongly induced in the articular joint of OA patients. We analyzed the gene expression of IL-1*β* and IL-6 after 6 h of stimulation with IL-1*β*, while the transcript levels of TNF*α* were studied after 1 h of stimulation. The MEC values for GS on all the inflammatory markers analyzed ranged between 1 and 10 *μ*M (Figure 1).

#### 3.1.2. GS Effect on Matrix Degradative Markers

**Figure 2:**
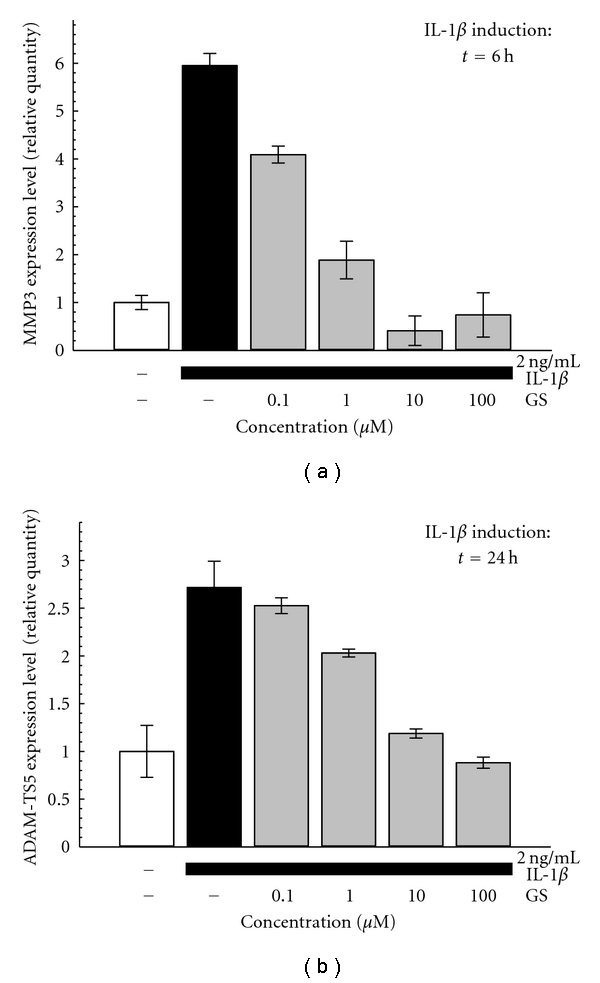
Effect of GS on IL-1*β*-induced gene expression of MMP-3 (a) and ADAM-TS5 (b). One hour after the pretreatment with GS (0,1–100 *μ*M), SW1353 are stimulated with IL-1*β* (2 ng/mL) as indicated. Representative pictures of 3 to 5 independent experiments are shown. Data are mean ± SD of three replicates.

We analyzed two extracellular matrix degradative markers commonly involved in the OA disease: a matrix metalloproteinase MMP-3 (stromelysin-1) and ADAM-TS5 (a disintegrin and metalloproteinase with thrombospondin motifs) also called aggrecanase 2. The analysis has been performed after 6 h of treatments for MMP-3 and after 24 h for ADAM-TS5. The MEC values for GS on matrix degradative markers analyzed ranged between 0.1 and 1 *μ*M (Figure 2).

#### 3.1.3. GS Effect on NF-*κ*B and AP-1 Subunits

**Figure 3:**
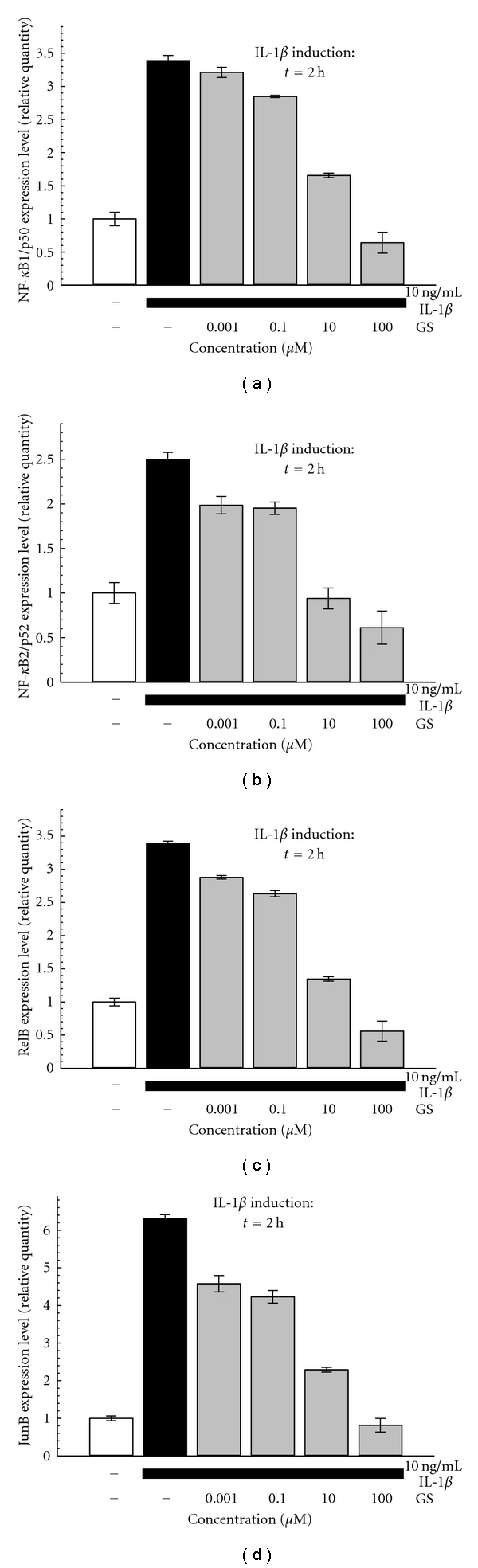
Effect of GS on IL-1*β*-induced gene expression of NF-*κ*B1/p50 (a), NF-*κ*B2/p52 (b), RelB (c), and JunB (d). One hour after the pre-treatment with GS (0,001–100 *μ*M), SW1353 are stimulated with IL-1*β* (10 ng/mL) for 2 h. Representative pictures of 3 to 5 independent experiments are shown. Data are mean ± SD of three replicates.

The gene expression analysis of NF-*κ*B subunits (p50, p52, and RelB) and of JunB was performed after 2 h of IL-1*β* stimulation. The MEC values for GS on transcription factors of the NF-*κ*B family members ranged between 1 nM and 0.1 *μ*M. JunB MEC value for GS was 1 nM (Figure 3).

### 3.2. *In Vivo* Results

**Figure 4:**
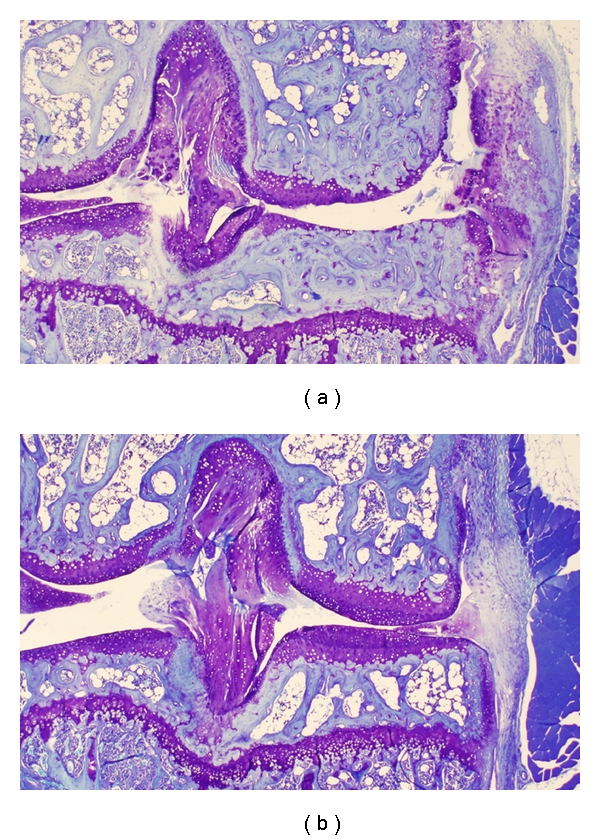
Representative histology images of articular cartilage from 8-month-old STR/ort mice that were treated with GS 200 mg/kg (b) or vehicle (a) for 3 months.

Eight-month-old STR/ort mice show a histological picture resembling that of human OA with a constant progression with age; the whole joint undergoes degenerative changes very much like human OA, that consist in articular cartilage peeling, clefting, fibrillation, erosion; subchondral bone sclerosis with osteocyte necrosis; focally, fatty involution of the epiphyseal bone marrow; synovial hyperplasia; and chondro-osseous metaplasias in capsula, ligaments, periosteum, leading to chondrophyte and osteophyte formation with ectopic bone development as depicted in Figure 4 (a).

**Figure 5:**
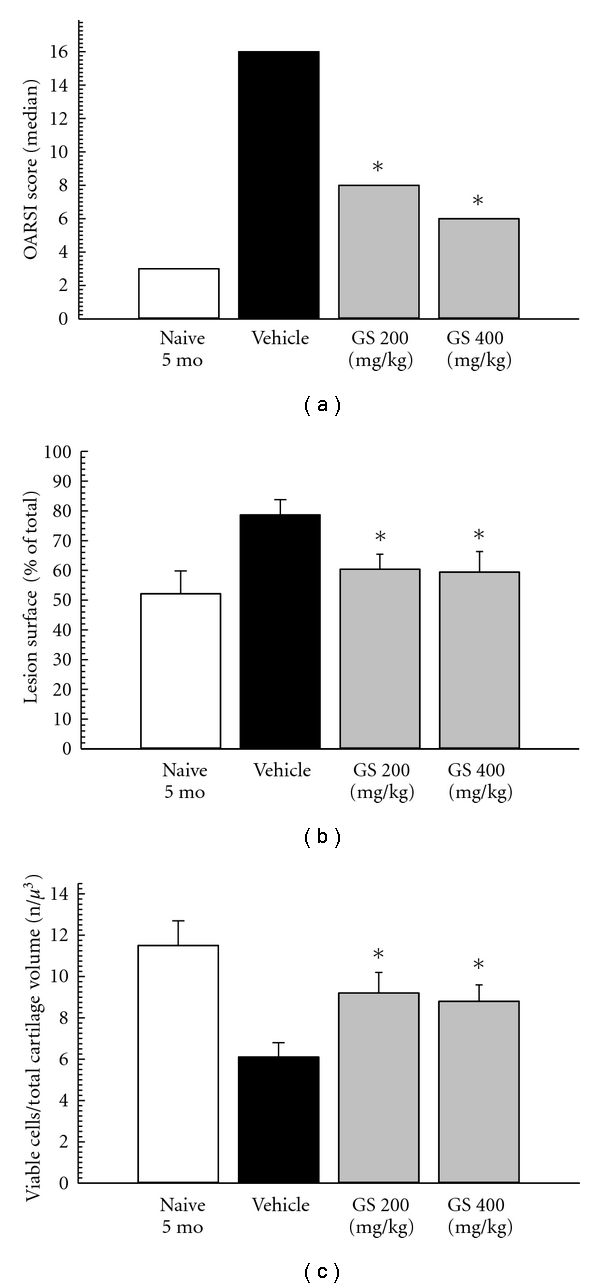
(a) Effect of subcutaneous administration of GS on OARSI score in STR/ort mice. Data are represented as median. (b) Effect of subcutaneous administration of GS on Lesion surface/total surface in STR/ort mice. Data are represented as mean ± SE. (c) Effect of subcutaneous administration of GS on n. of viable cells/total cartilage volume in STR/ort mice. Data are represented as mean ± SE. * = *P* < 0.05 versus vehicle (ANOVA). The vehicle group is always statistically different from the naïve 5-month-old group.

In this relevant animal model of spontaneous OA, we observed that all OA scores were significantly decreased following treatment with GS compared to vehicle (Figure 4(b), Figure 5(a) and data not shown). In particular, the OARSI score takes into account both the depth of the lesion on the articular surface, and its width. We observed that both doses of GS substantially and significantly improved the OARSI score. A histomorphometrical analysis was also performed. We observed that all parameters tended to an improvement following treatment with GS; in particular, the lesion surface on the articular surface was significantly decreased (Figure 5(b)) and the number of live chondrocytes within the articular cartilage matrix (viable cells/total cartilage volume) significantly increased (Figure 5(c)) in both GS groups compared to vehicle.

## 4. Discussion

### 4.1. Findings in Cell Biology

The mechanism of action beneath the favorable actions of GS has not yet been fully elucidated; however, evidence is available that deserves to be discussed. To understand the molecular site(s) of action of glucosamine, several *in vitro* studies have been performed demonstrating different relevant activities using concentrations in the range of 1 *μ*M–1 mM. Largo and coworkers [18] indeed demonstrated that GS can inhibit NF-*κ*B activity as well as the nuclear translocation of p50 and p65 proteins in cultures of human osteoarthritic chondrocytes stimulated with IL-1*β*. These observations allow us to postulate the involvement of NF-*κ*B in GS's mechanism of action, although GS was used in a concentration range between 0.2 and 2 mM, much higher than that found in the plasma by Persiani et al. after GS administration [12]. A very recent paper demonstrated that, at least in rabbits, plasma levels of glucosamine appeared to be well correlated with cartilage concentrations, being, therefore, useful to predict the target cartilage concentration and its pharmacological activity [19]. On the other hand, Chan et al. [20] showed that glucosamine at these clinically relevant concentrations (about 20 *μ*M) reduced COX-2, iNOS, and mPGEs1 gene expression and PGE_2_ synthesis after IL-1*β* stimulation, suggesting that also in this range of concentration Glucosamine can control the cascade triggered by inflammatory stimuli. Of late, observations have been published that point out the blunting effect of Glucosamine on NF-*κ*B-dependent transcription via an epigenetic mechanism [21].

IL-1*β* is a potent proinflammatory cytokine produced in high amounts in the OA joint [22, 23], where it induces a series of gene expression alterations. This cytokine triggers the expression of inflammatory factors such as COX-2, iNOS, IL-6, IL-1*β*, TNF*α*, and matrix degradation factors, namely, MMPs and ADAM-TSs. Most of these genes are under transcriptional control of the nuclear factor *κ*B (NF-*κ*B). NF-*κ*B is a collective name for homo- and hetero-dimeric complexes of Rel family polypeptides, which are present, in mammals, with five members: RelA (p65), RelB, c-Rel, NF-*κ*B1/p50, and NF-*κ*B2/p52. In unstimulated cells, the majority of NF-*κ*B dimers are retained in the cytoplasm as an inactive complex bound to inhibitor proteins (I*κ*Bs). In response to different activating stimuli, including IL-1*β*, I*κ*Bs are degraded proteolitically by the 26S proteasome, after phosphorylation by IKK and polyubiquitination. NF-*κ*B dimers then translocate to the nucleus and bind to specific consensus sequences (*κ*B elements) along the DNA, promoting gene transcription. To clarify the mode of action of GS at therapeutically achievable concentrations we chose an appropriate human chondrocyte model, the human chondrosarcoma cell line SW1353 [24–26]. Moreover, as well depicted by Block et al. [11], glucose concentration in the medium is one of the major confounding factors for the interpretation of in vitro experiment results reported in the literature. It is known that most of the published in vitro studies with glucosamine have been performed in culture medium containing 25 mM of glucose, that could easily compete with glucosamine for the ubiquitous sodium-independent facilitative glucose transporter GLUT1 impeding efficient glucosamine uptake into cells. Therefore, our experiments in SW1353 cells were performed in Leibovitz medium containing 5 mM D-galactose a less efficient substrate for GLUT-1 [27] instead of D-glucose. Under these physiologically relevant conditions, we employed quantitative RT-PCR to study GS's activity in counteracting the effects of IL-1*β* on the expression of several genes relevant for inflammation and matrix metabolism. We demonstrated that GS sulfate is effective in a dose-dependent manner on modulating OA-relevant gene expression triggered by IL-1*β*, also at the low concentrations (1–10 *μ*M) found in human pharmacokinetic studies. These events are probably initiated by a decrease in NF-*κ*B translocation. Indeed, Letari et al. [28] showed by EMSA that the increase in nuclear NF-*κ*B triggered by IL-1*β* was blunted by GS, and the effect on transcription might be sustained thereafter by the inhibition of NF-*κ*B subunit expression. Of note, these observations are in agreement with the studies discussed above [18, 20, 21, 29].

### 4.2. Histopathological and Further *In Vivo* Outcomes

We have tried to provide some contribution to a better understanding of the effect of GS at a tissue level. In the search for reliable and predictive animal models of OA to use for drug discovery purposes, we considered that surgical OA in the animal may not necessarily reflect all aspects of spontaneous idiopathic OA in the elderly; therefore, we resolved to also take advantage of a mouse strain that is believed to be a relevant model of human OA, the STR/ort mouse (reviewed by Mason et al. [14]). The pathological features of this particular mouse strikingly recapitulate several characteristics of human OA patients. In this model, we observed that GS improved the pathological severity and the histological parameters of OA. These observations are consistent with the clinical effect of GS. 

Current methods employed in the clinics to assess efficacy of compounds aimed at modifying the course of OA do not allow for a detailed investigation of the effects of such compounds at a tissue level. To this end, the use of animal models of OA has represented a very important and enlightening instrument, and indeed discussion has been going on for long on what models are most representative of human OA, and predictive. Surgical models employing various rodent and nonrodent species are definitely the most studied and used so far.

An early study with GS was that of Altman and Cheung [30]. Partial meniscectomy was performed to adult rabbits that were then treated with GS 100 or 200 mg/kg /die or vehicle for 12 weeks. Histology showed that rabbits treated with vehicle suffered severe fibrillation and clefting of the articular cartilage accompanied by chondrocyte loss at the operated knee; treatment with GS significantly prevented those degenerative changes to a remarkable extent. Expression of various MMPs was also investigated by immunostaining and found increased in the vehicle but not in the GS groups.

A limit of that study was that of a preventive treatment, that is, knowing when the injury occurred, treatment was started immediately. That situation does not necessarily mimic the human condition, where disease develops spontaneously and progressively and starts to be treated when already advanced to some degree. 

The same criticism may not apply to another study, that of Tiraloche et al. [31] in which a different surgical protocol in rabbits was applied, transection of the anterior cruciate ligament (ACLT), and in which treatment with Glucosamine hydrochloride (100 mg/die) was started 3 weeks after surgery. The histopathological features observed were parallel to those reported above. In this study, Glucosamine hydrochloride failed to produce a significant improvement in most of the OA parameters evaluated, except one, that is, GAG loss. However, it must be considered that Glucosamine hydrochloride has poorer pharmacokinetics than GS [12, 13, 32, 33].

A very recent work yet again demonstrated efficacy of GS in a different animal model, in which a true curative protocol was employed [29]. These authors performed ACLT in adult rats, and then allowed OA to develop for five weeks before starting treatment. The authors then provided an impressive array of readouts, that included assessment of nociception (mechanical allodynia, weight bearing distribution test) and macroscopic and histopathologic evaluations of tissue degeneration. In all of these measurements, GS (250 mg/kg/die) had a significant effect in reducing the severity of OA, both in terms of pain and of structural integrity of the tissues involved. Furthermore, the authors have also provided hints to a possible cellular signaling pathway involved in the molecular action(s) of GS. They observed that both p38 MAPK and JNK, two key intracellular mediators of inflammatory signals, were activated following OA establishment, and that treatment with GS hampered these activations. Such observations are in agreement with evidence coming from cell culture experiments, which are discussed above.

### 4.3. Conclusion

Evidence concerning GS's mechanism of action has been shedding some light on how GS exerts its effects. It appears now from several studies, including ours, that GS inhibits gene expression of different inflammation and matrix degradation markers, at concentrations similar or even lower than that found in human plasma after oral therapeutic doses, by interfering with the NF-*κ*B pathway. It is definitely likely that there is more than that as to what GS does within the cell, and that much remains to be established. At the same time, to the best of our knowledge this is the first study that describes in detail the effects of GS at a tissue level in improving articular cartilage health in a spontaneously occurring OA model that recapitulates so many aspects of human idiopathic OA in the elderly.
